# Hepatic hydatid cyst fistulized in the duodenum: Extremely rare complication

**DOI:** 10.1016/j.radcr.2021.09.038

**Published:** 2021-10-16

**Authors:** Hatim Essaber, Youssef Ihab, Leila Jroundi, Fatima Ezzahra Laamrani

**Affiliations:** a Service de la radiologie des urgences, CHU Ibn Sina, Rabat, Morocco; b Service urgences chirurgicales viscérales, CHU Ibn Sina, Rabat, Morocco

**Keywords:** Hepatic Hydatid cyst, Fistulized, Duodenum

## Abstract

The fistulization of a hepatic hydatid cyst to the duodenum is an extremely rare case demonstrated in computed tomography and confirmed surgically. We reported a case of 56 years-old woman representing this unusual complication.

We show, through this work, the importance of radiological signs that can help to make the diagnosis before surgery.

## Introduction

Hepatic hydatid cyst is a zoonotic disease commonly caused by Echinococcusgranulosus in cattle and sheep rearing regions.

It is endemic in countries around the Mediterranean, such as Morocco, and constitutes a real public health problem due to its frequency, morbidity and potential mortality.

Numerous complications can occur, including compression, infection, or rupture.

We see various types of ruptures (in the bile ducts, into the peritoneal, pleural cavity) but into the gut, especially the duodenum, still extremely rare and challenging for the surgeon.

### Clinical history

A 56-years-old woman with a history of asymptomatic hepatic hydatid cyst came to the emergency with fever, sweating and right upper quadrant pain that increased with inspiration for 2 days.

Laboratory studies revealed a white blood cell count of 13 300/μl, with 37% eosinophils. Biochemistry revealed a creatinine level of 140 μmol/l.

CT scan showed a hepatic cavity occupying segment 6 at his lower pole; we observed a fistula communicating with the duodenum. A second sign that testifies this communication was the air-liquid level.

Emergency laparotomy was carried out under the diagnosis of an infected hydatid cyst. The patient was operated on through a bilateral subcostal laparotomy and finding 1 hydatid cyst that is close to the pylori but with direct contact with the first duodenum knee.

**Fig. 1:**
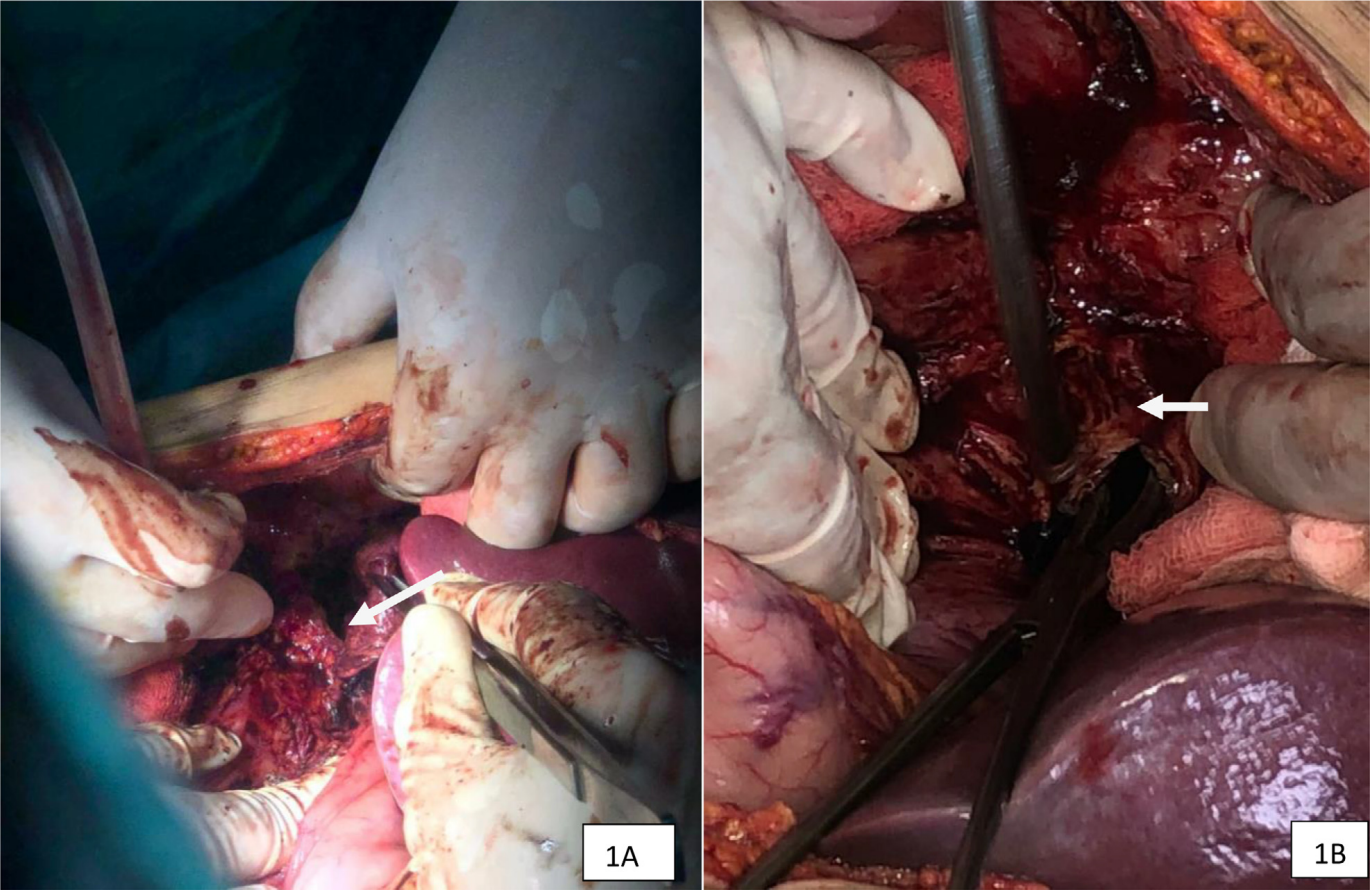
(A and B): Pictures token in per operation; the resection of the hydatid cyst and repair of the duodenal wall defect The white arrow in the picture (A) demonstrate the exploration of the cavity after ablation of the hepatic hydatid cyst. The white arrow in the picture (B) show the management of the fistula found communicating with the duodenum.

Exploring this cystic cavity revealed communication with the first duodenum affecting its anterior wall, which was repaired and the hydatid cyst removed (Figs. 1A and B).

## Discussion

Hydatidosis is a parasitic disease considered benign but can become dangerous because of its complications. It is a real public health problem in the world by its frequency, morbidity, and its potential mortality. It is caused by infection with the larval stage of Echinococcusgranulosus.

Many serious complications dominated by rupture and secondary infection [1], [2], [3], [4].

We consider that the rupture is the most notable complication of hepatic hydatid cyst (about 15% of all cases) [1,2]with high risk of death [4].

The rupture is caused by 2 major factors the inflammation, the diameter of the cyst, the permanent contact with is and the delay of the treatment lead to erosion of the wall of the organ adjacent to the cyst.

It usually occurs into biliary ducts (9%-30% in large series), causing jaundice and acute cholangitis, or into the peritoneal cavity (1%-12.5%), which might lead to a life-threatening anaphylactic reaction [5].

Only a few cases in literature talk about the rupture or fistulisation of the hydatid cyst into the duodenum (0.15%), and its diagnosis is usually after surgery.

The presence of air in the cyst was observed in most of the cases in the literature, which may alert the physician to fistulisation formation, but is not a specific sign.

**Fig. 2:**
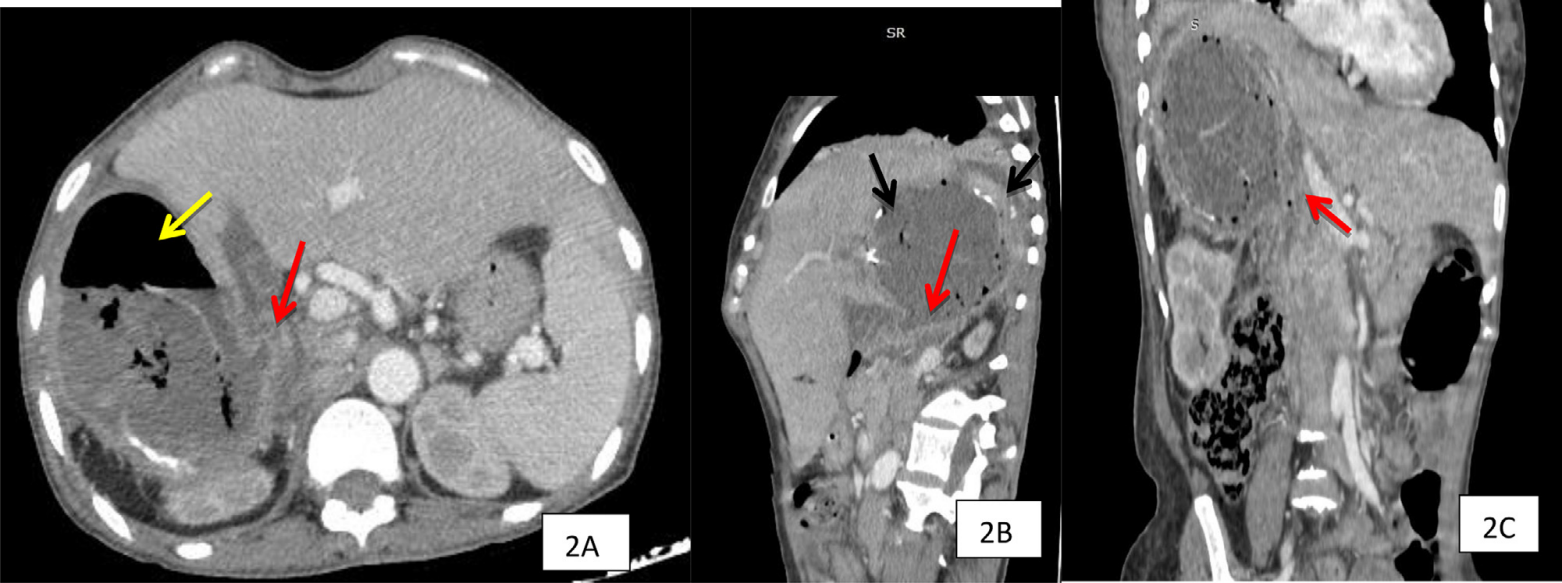
CT scan of the hepatic area with contrast in portal phase, axial (A) sagittal (B), and coronal (C) images show a hydatid cyst of the right hepatic lobe with air-fluid level (yellow arrow) and calcified walls (black arrows). They are Demonstrating also the communication between the duodenum and hepatic cyst (red arrows) (color version of figure is available online).

However, in our case and without barium opacification, we can identify the fistula target communicating with the first knee of the duodenum, and at the same time, the cyst contains an air-fluid level (Figs 2 A, B and C).

Nowadays, interventional radiological treatment takes place, especially for stable patients that cannot support surgery. However, we can propose conservative surgical treatments such as partial cystectomy or drainage.

The surgical management depends essentially on the view of hollow viscus damage, in other words, the status of the defect if it is a small gap or not and if it is revamped by the local inflammatory condition.

In terms of what is present, the surgical procedure to log out the cysto-digestive fistula by simple suture associated with gastric aspiration or other options such as duodenostomy or duodenal diverticulization if the duodenal gap was important and would not lend itself to reparation.

Radical surgical procedures like hepatic resection or pericystectomy can also be performed in selected complicated cases [6].

Treatment of the parasite by preoperative and postoperative albendazole effectively prevents recurrence, and the recurrence rate after all surgical treatment alternatives is less than 10% [7].

## Conclusion

The lesson learned from this case study is to look for the clue findings in order to make the diagnosis of the fistulisation of the hepatic hydatid cyst which is extremely rare and always challenging for the surgeon.

## References

[1] Lo Casto A, Salerno S, Grisanti M, Mastrandrea G. (1997). Hydatid cyst of the liver communicating with the left colon. J Radiol.

[2] Diez Valladares L, Sanchez-Pernaute A, Gonzalez O, Perez-Aguirre E, Talavera P, Gutierrez del Olmo A (1998). Hydatid liver cyst perforation into the digestive tract. Hepatogastroenterology.

[3] Nouira K, Hila H, Bedioui H, Baccar S, Ben Messaoud M (2007). Rupture of hydatid cyst of the liver into the duodenum: a case report. Tunis Med.

[4] Noguera M, Alvarez-Castells A, Castella E, Gifre L, Andreu J (1993). Spontaneous duodenal fistula due to hepatic hydatid. Abdom Imaging.

[5] Akcan A, Sozuer E, Akyıldız H, Ozturk A, Atalay A, Yılmaz (2010). Predisposing factors and surgical outcame of complicatted liver hydatid cysts. Word J Gastroenterol.

[6] Botrugno I, Gruttadauria S, Li Petri S, Davide Cintorino, Marco Spada, Fabrizio Di Francesco, Dullio Pagano (2010). Complex hydatid cyst of the liver: a single center's envolving approach to surgical treatement. Am Surg.

[7] Secchi MA, Pettinari R, Mercapide C, Ricardo Bracco, Carlos Castilla, Eduardo Cassone, Pablo Sisco (2010). Surgical management of liver hydatidosis: a multicentre series of 1412 patients. Liver Int.

